# Effectiveness of proximal tibial tubercle transfer in patients with *patella baja* after total knee arthroplasty

**DOI:** 10.1186/s40634-022-00452-4

**Published:** 2022-02-15

**Authors:** Ines Unterfrauner, Ruben Loretz, Nathalie Kühne, Florian Grubhofer, Sandro F. Fucentese

**Affiliations:** 1 Department of Orthopedics, Balgrist University Hospital, University of Zurich, Forchstrasse 340, 8008 Zurich, Switzerland; 2 Unit of Clinical and Applied Research, Balgrist University Hospital, University of Zurich, Forchstrasse 340, 8008 Zurich, Switzerland

**Keywords:** Proximal tibial tubercle transfer, Tibial tubercle proximalisation, Tuberositas osteotomy, *Patella baja*, Patellar height, Total knee arthroplasty, Arthrofibrosis, Functional outcome

## Abstract

**Purpose:**

*Patella baja* after total knee arthroplasty (TKA) is a common problem that is usually treated via proximal transfer of the tibial tubercle. As the long-term outcomes of this procedure are unclarified, this study aimed to investigate the changes in clinical function and radiographic patellar height during five years of follow-up.

**Methods:**

Sixty patients with *patella baja* after TKA who underwent proximalisation of the tibial tubercle were followed up for a mean of 71 months (range 21–153 months). The pre- and postoperative range of motion (ROM) and clinical scores (Knee Society Score (KSS), Western Ontario and McMaster Universities Osteoarthritis Index (WOMAC)) were compared. The radiographic patellar height was measured with the Caton-Deschamps index (CDI), Blackburne-Peel ratio (BP), and modified Insall-Salvati index (MIS).

**Results:**

Proximalisation of the tibial tubercle resulted in a significant improvement in the ROM from 80° to 88°. The KSS and WOMAC did not improve or even worsened after the intervention. The radiographic patellar height immediately after tibial tubercle transfer was not better than prior to the intervention (CDI 0.72 vs. 0.63, *p* = 0.72; BP 0.66 vs. 0.61, *p* = 0.72; MIS 1.59 vs. 1.55, *p* = 1.00) and further decreased significantly so that the mean final values were worse than the values in the native joint (CDI 0.59 vs. 0.78, *p* = 0.001; BP 0.58 vs. 0.74, *p* = 0.001; MIS 1.39 vs. 1.81, *p* < 0.001).

**Conclusion:**

Proximalisation of the tibial tubercle in patients with *patella baja* after TKA does neither lead to significant improvements in the clinical outcome nor in the radiographic patellar height during long-term follow-up.

**Level of evidence:**

III

## Introduction

The patient-reported rate of satisfaction at five years after total knee arthroplasty (TKA) is around 75% [3], and patella-related problems are the cause of dissatisfaction in half of these cases [2]. In addition to patellar maltracking, instability, fractures, and impingement [11], patellar malposition constitutes a common pathology that appears in 34% of patients and results in anterior knee pain and impaired range of motion (ROM) [10, 16]. Whilst true *patella baja *occurs due to scarring and shortening of the patellar tendon, pseudo-*patella baja* is associated with an elevation of the joint line because of tibial under-resection or femoral over-resection by preservation of the patellar tendon length [6].

Although the literature is limited, proximal transfer of the tibial tubercle is regarded as a satisfactory and logical solution for *patella baja* after TKA [4, 5, 14, 15]. This procedure was first described by Dolin in 1983 as a method to gain better articular exposure in patients undergoing TKA by performing a coronal osteotomy of the tibial tubercle [7]. The few studies of the mid-term outcomes of proximalisation of the tibial tubercle report pain relief [8, 15] and functional improvement [8, 14, 15]. However, although previous studies report radiographic normalisation of the patellar height after proximalisation of the tibial tubercle [8, 15], we have observed insufficient ROM in our patients after proximal transfer of the tibial tubercle and suspected that the patellar height underwent a de novo decline over time after the initial improvement. To the best of our knowledge, no study has investigated the long-term radiographic patellar height and functional outcome of proximal transfer of the tibial tubercle for *patella baja* after TKA. Therefore, the aim of this study was to evaluate the long-term clinical function of the knee and to investigate the long-term radiographic change in the patellar height after proximalisation of the tibial tubercle due to *patella baja* after TKA.

## Materials and methods

### Study cohort

After the study protocol was approved by the State Ethical Committee (BASEC 2019–02273; Appendix 1), the database of Balgrist University Hospital was screened to construct the study cohort. The study cohort comprised 60 patients with *patella baja* after TKA who underwent revisional proximalisation of the tibial tubercle at our tertiary centre for knee surgery from February 2007 to August 2018. Patients who underwent proximalisation of the tibial tubercle for any reason other than *patella baja* (for example, instability) were excluded. The study cohort was invited to undergo clinical and radiological follow-up at our institution. From all 60 patients invited for a follow-up control, 34 individuals were available for another examination after a mean of 71 months (range 21–153 months) and provided informed consent for study participation. For the remaining 26 patients who were not available for follow-up examination, the last available radiographs and clinical data were used for further analysis. Thereby, eight patients were lost to follow-up within the first year after surgery. Hence, the overall mean follow-up time was 37 months (1–142 months). The whole study cohort had a mean age of 61.4 years (range 34–82 years) and average BMI of 29.9 kg/m^2^ (range 19.8–50.6 kg/m^2^), and 50% were women (Table 1).

**Table 1:** Demographic data of the study cohort

Variable	*n* = 60	Number of missing values
Age	61.4 ± 10.6 (34–82)	
Female sex	30 (50)	
BMI	29.87 ± 5.89 (19.82–50.64)	
Native CDI	0.78 ± 0.14 (0.47–1.05)	29
Pre-TO CDI	0.63 ± 0.21 (0.12–1.03)	4
Post-TO CDI	0.72 ± 0.23 (0.22–1.24)	
Last CDI	0.59 ± 0.23 (0.21–1.23)	
Native BP	0.74 ± 0.13 (0.49–1.00)	29
Pre-TO BP	0.61 ± 0.21 (0.11–1.07)	4
Post-TO BP	0.66 ± 0.20 (0.22–1.07)	
Last BP	0.58 ± 0.23 (0.20–1.23)	
Native MIS	1.81 ± 0.28 (1.27–2.32)	29
Pre-TO MIS	1.55 ± 0.35 (0.76–2.15)	4
Post-TO MIS	1.59 ± 0.41 (1.00–2.83)	
Last MIS	1.39 ± 0.38 (0.68–2.30)	
Pre-TO ROM	80 ± 25 (20–125)	2
Last ROM	88 ± 26 (5–125)	
Pre-TO KSS Knee Score	51.6 ± 16.4 (8–78)	40
Last KSS Knee Score	56.3 ± 20.6 (18–94)	4
Pre-TO KSS Function Score	65.5 ± 20.1 (0–90)	40
Last KSS Function Score	60.0 ± 18.4 (0–90)	4
Pre-TO WOMAC	4.5 ± 1.4 (2.0–6.4)	48
Last WOMAC	4.4 ± 2.0 (0.0–7.6)	26

Continuous variables are shown as mean ± standard deviation (range)

Categoric variables are presented as number (percentage)

*CDI* Caton-Deschamps index, *BP* Blackburne-Peel ratio, *MIS* modified Insall-Salvati index, *ROM* range of motion, *KSS* Knee Society Score, *native* before total knee arthroplasty, *pre-TO* immediately before tuberositas osteotomy, *post-TO* immediately after tuberositas osteotomy, *last* final follow-up

### Surgical technique

The average interval between TKA and proximalisation of the tibial tubercle was three years (range 0–16 years). Eleven (18%) of 60 patients underwent closed manipulation under anaesthesia prior to proximalisation of the tibial tubercle.

**Fig. 1:**
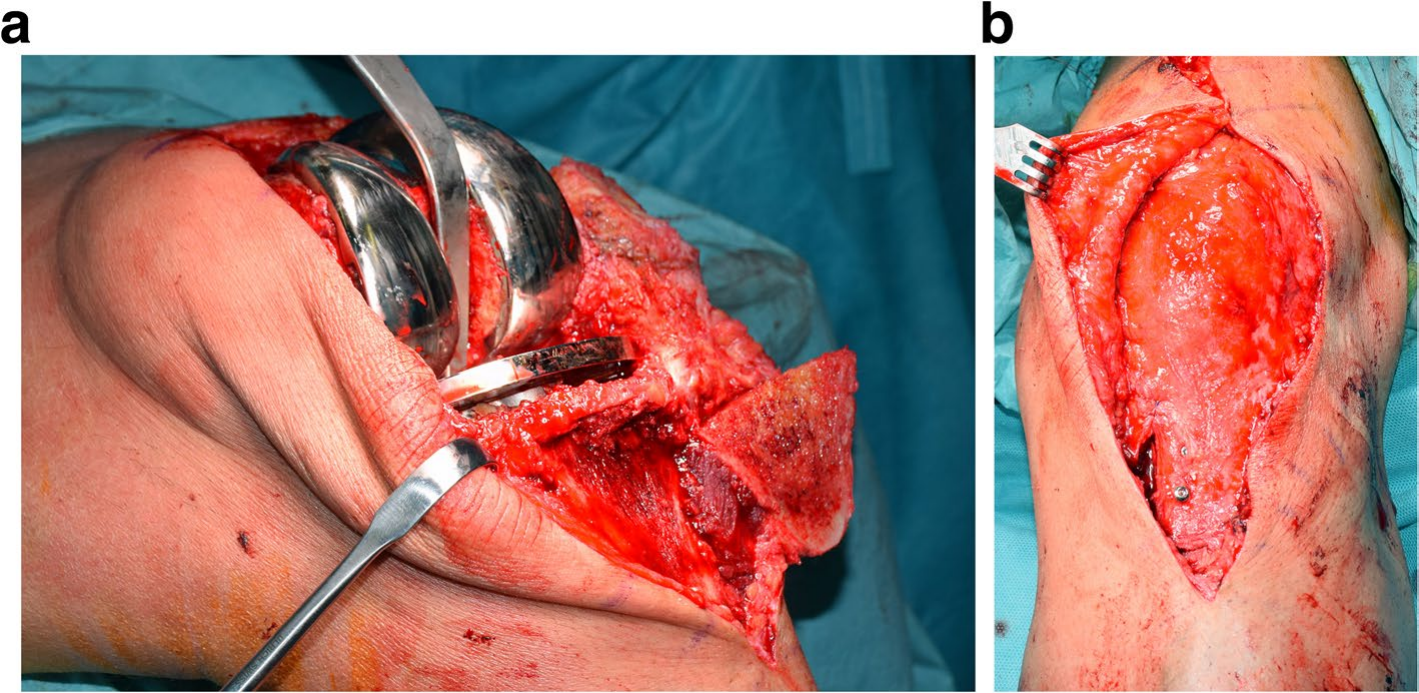
**a** Intraoperative presentation of the osteotomy of the tibial tubercle with TKA from the medial side of the anterior edge of the tibial tubercle. **b** Intraoperative presentation of fixation of osteotomised tibial tubercle with lag screws

The patients were placed in supine position under general or spinal anaesthesia with a tourniquet applied. The skin incision was extended distally, and the patellar ligament was visualized and protected. The 4–6-cm-long osteotomy was performed from the medial side of the anterior edge of the tibial tubercle (Fig. 1a). A bone bridge of at least 8 mm from the posterior side of the tibial component was preserved to avoid destruction of the bone-cement-prosthesis interface; this was the largest degree of tubercle proximalisation. The osteotomised tibial tubercle was secured with three 3.5-mm lag screws (Fig. 1b). After proximalisation, the ROM and patellar tracking were checked; in six out of the 60 cases (10%), a lateral retinaculum release was performed to correct maltracking. The arthrotomies were closed and drainage was inserted. The postoperative procedure included partial weightbearing with 15 kg for six weeks postoperatively, with avoidance of forced knee extension against resistance and a free ROM.

All interventions were performed by three senior orthopaedic surgeons. Besides proximalisation of the tibial tubercle, 25 (42%) of 60 patients underwent one-stage revision of the knee prosthesis, whereas four (7%) received two-stage revision arthroplasty because of periprosthetic infection and 37 (62%) underwent additional change of the inlay due to confirmed or suspected infection. Furthermore, 16 patients (27%) with femoropatellar pain and corresponding signs of degeneration on the retropatellar surface underwent simultaneous patellar resurfacing and tibial tubercle transfer, while three (5%) underwent patellar resurfacing secondary to tibial tubercle proximalisation.

### Clinical outcome

The total active ROM (measured with a goniometer) and clinical scores (Knee Society Score (KSS) and Western Ontario and McMaster Universities Osteoarthritis Index (WOMAC)) prior to proximalisation of the tibial tubercle were extracted from the patient records. These clinical outcomes were reassessed at final follow-up.

### Radiographic variables

**Fig. 2:**
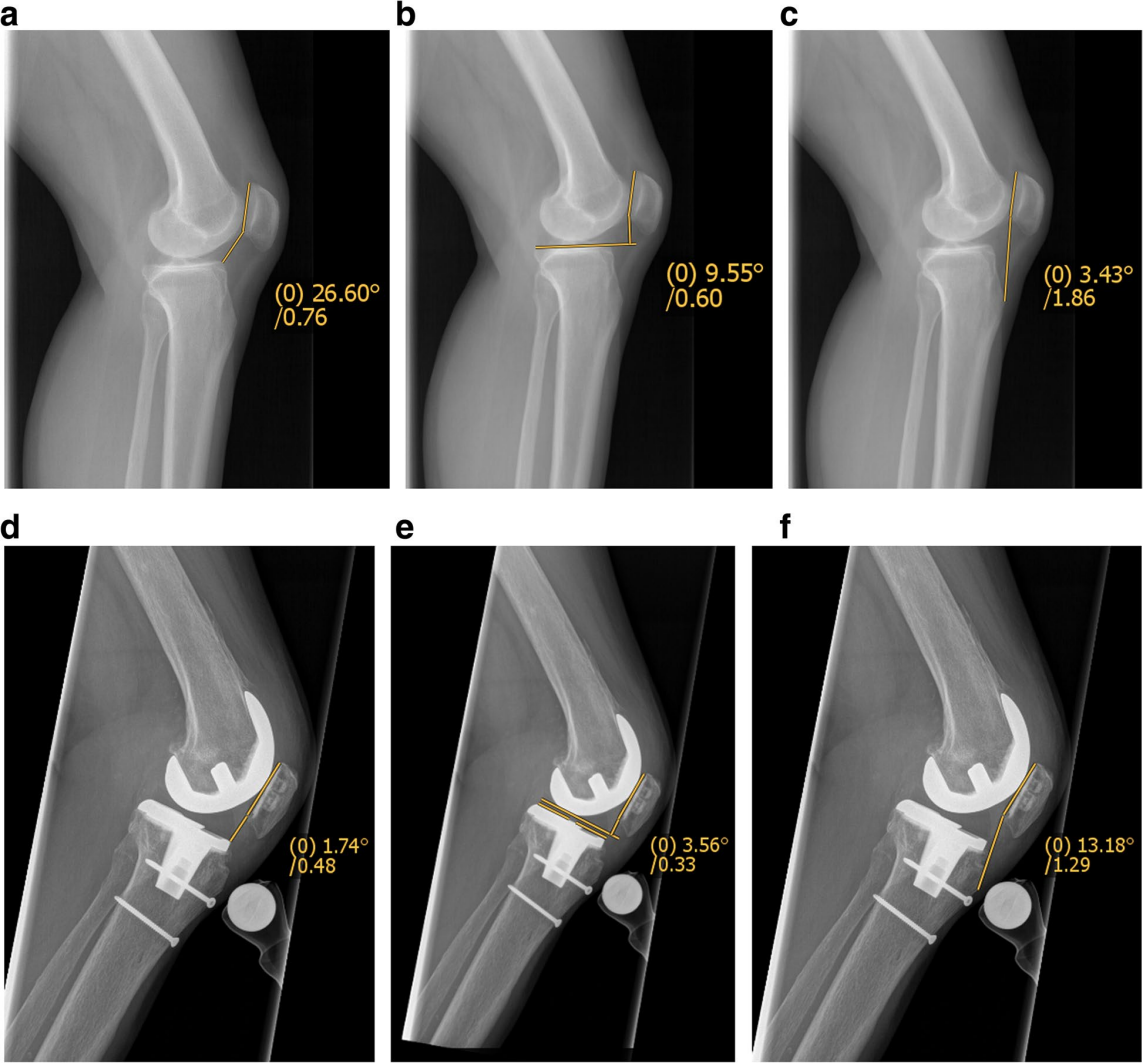
**a** Measurement of the Caton-Deschamps index (CDI) in the left native knee joint of a 63-year-old woman. **b** Measurement of the Blackburne-Peel ratio (BP) in the left native knee joint of a 63-year-old woman. **c** Measurement of the modified Insall-Salvati index (MIS) in the left native knee joint of a 63-year-old woman. **d** Measurement of the Caton-Deschamps index (CDI) in the left knee joint of a 63-year-old woman with TKA. **e** Measurement of the Blackburne-Peel ratio (BP) in the left knee joint of a 63-year-old woman with TKA. **f** Measurement of the modified Insall-Salvati index (MIS) in the left knee joint of a 63-year-old woman with TKA

To radiologically investigate the patellar tendon length and patellar height and identify *patella baja*, the Caton-Deschamps index (CDI) [4], Blackburne-Peel ratio (BP) [1], and modified Insall-Salvati index (MIS) [9] were measured on lateral radiographs of the 30° standardised flexed knee at four timepoints: in the native joint prior to TKA (native), immediately before proximalisation of the tibial tubercle (pre-TO), immediately after proximalisation of the tibial tubercle (post-TO), and at final follow-up (last) (Figs. 2a-c in the native joint, Figs. 2d-f in case of TKA). Whereas the BP and CDI both assess patellar height, the MIS calculates the ratio between the length of the patellar tendon and the patella itself. Therefore, the CDI and BP have low intra- and interobserver variations and low standard error [12]. All radiographic variables were measured by two orthopaedic surgeons (IU, RL), and the interobserver reliability was analysed.

### Statistical analysis

The primary outcomes were the changes in the ROM, clinical scores (KSS and WOMAC), and radiographic values (CDI, BP, and MIS) between the native, pre-TO, post-TO, and last timepoints. The secondary outcome was the correlation between the clinical outcomes and radiographic variables.

Groups (based on various timepoints) were compared using the Wilcoxon-rank-sum test for continuous variables, and the Kruskal-Wallis test for continuous variables; post-hoc analysis was performed with the Dunn-Bonferroni test.

Spearman correlation coefficients were used to measure correlations between the clinical and radiographic variables. IBM SPSS Statistics 25 was used. Differences were considered significant when the two tailed *p* value was less than 0.05; correlations were considered moderate when the correlation coefficient exceeded 0.30, and strong when the correlation coefficient exceeded 0.6.

## Results

### Clinical outcomes

**Fig. 3:**
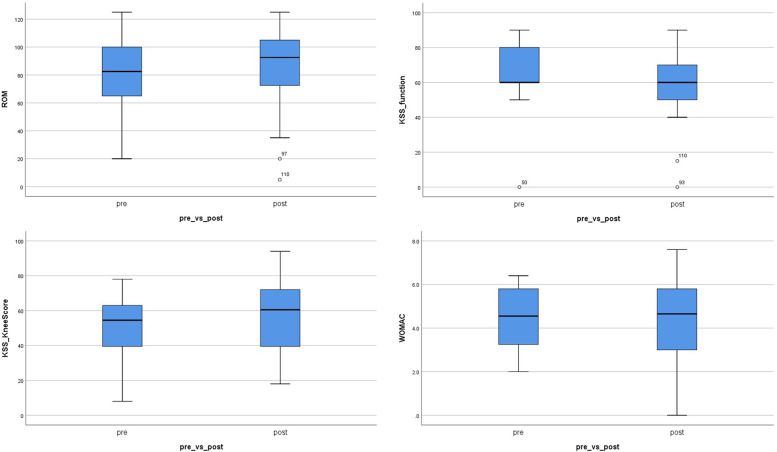
Comparison of the range of motion (ROM), Knee Society Score (KSS), and Western Ontario and McMaster Universities Osteoarthritis Index (WOMAC) before and after proximalisation of the tibial tubercle

Among the 60 patients with *patella baja* after TKA, more than one third had a knee flexion of less than 75°. Compared with the pre-TO ROM, the total active ROM at last follow-up significantly improved to a mean value of 88° (Table 2, Fig. 3). Overall, 32 of 60 patients had improved ROM after tibial tubercle transfer; the ROM after proximalisation was more than 100° in 25 of 60 patients, with one patient achieving a ROM of 125°.

**Table 2:** Intergroup comparisons of clinical outcomes before and after proximalisation of the tibial tubercle

Variables	Pre-TO	Last	*p* value
ROM	80 ± 25 (20–125; 2 missing)	88 ± 26 (5–125; 0 missing)	***0.037***
KSS Knee Score	51.6 ± 16.4 (8–78; 40 missing)	56.3 ± 20.6 (18–94; 24 missing)	0.13
KSS Function Score	65.5 ± 20.1 (0–90; 40 missing)	60.0 ± 18.4 (0–90; 24 missing)	0.16
WOMAC	4.5 ± 1.4 (2.0–6.4; 48 missing)	4.4 ± 2.0 (0–7.6; 26 missing)	0.21

Continuous variables are shown as mean ± standard deviation (range; number of missing values)

Significant differences are displayed in **bold and italic**

*ROM* range of motion, *KSS* Knee Society Score, *pre-TO* immediately before tuberositas osteotomy, *last* final follow-up

The average KSS knee score tended to improve, but did not significantly change after the intervention. Both the KSS function score and WOMAC were worse at the last compared with the pre-TO timepoint (Table 2, Fig. 3).

Patients with a postoperative ROM of more than 100° showed a higher average improvement in all clinical scores between the pre-TO and last timepoints, whereas those with a postoperative ROM below 100° showed worsening of the clinical scores between the pre-TO and last timepoints.

### Radiographic variables

**Fig. 4:**
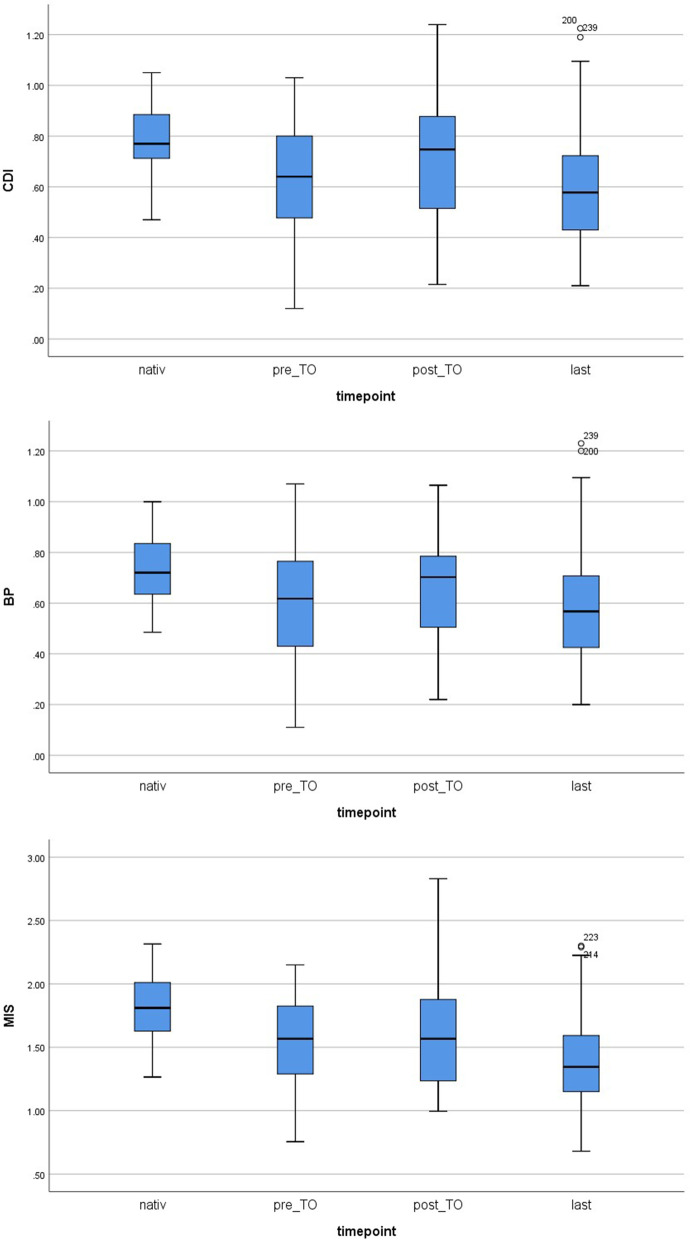
Comparison of the Caton-Deschamps index (CDI), Blackburne-Peel ratio (BP), and modified Insall-Salvati index (MIS) at various timepoints

All radiographic variables showed similar trends over time (Table 3, Fig. 4). The mean post-TO CDI and BP were not significantly better than the pre-TO values (CDI 0.72 vs. 0.63, *p* = 0.72; BP 0.66 vs. 0.61, p = 0.72) and even decreased over time so that the mean last CDI and BP values were significantly worse than the native values (CDI 0.59 vs. 0.78, *p* = 0.001; BP 0.58 vs. 0.74, p = 0.001) (Tables 3 and 4). Likewise, the post-TO MIS was similar to the pre-TO value (1.59 vs. 1.55, *p* = 1.00) and decreased further over time so that the mean last MIS was significantly lower than the native MIS (1.39 vs. 1.81, *p* < 0.001) (Tables 3 and 4).

**Table 3:** Intergroup comparisons of radiographic variables at various timepoints

Variables	Native	Pre-TO	Post-TO	Last	*p* value
CDI	0.78 ± 0.14 (0.47–1.05; 29 missing)	0.63 ± 0.21 (0.12–1.03; 4 missing)	0.72 ± 0.23 (0.22–1.24; 0 missing)	0.59 ± 0.23 (0.21–1.23; 0 missing)	***< 0.001***
BP	0.74 ± 0.13 (0.49–1.00; 29 missing)	0.61 ± 0.21 (0.11–1.07; 4 missing)	0.66 ± 0.20 (0.22–1.07; 0 missing)	0.58 ± 0.23 (0.20–1.23; 0 missing)	***0.001***
MIS	1.81 ± 0.28 (1.27–2.32; 29 missing)	1.55 ± 0.35 (0.76–2.15; 4 missing)	1.59 ± 0.41 (1.00–2.83; 0 missing)	1.39 ± 0.38 (0.68–2.30; 0 missing)	***< 0.001***

Continuous variables are shown as mean ± standard deviation (range; number of missing values)

Significant differences are displayed in **bold and italic**

*CDI* Caton-Deschamps index, *BP* Blackburne-Peel ratio, *MIS* modified Insall-Salvati index, *native* before total knee arthroplasty, *pre-TO* immediately before tuberositas osteotomy, *post-TO* immediately after tuberositas osteotomy, *last* final follow-up

**Table 4:** Dunn-Bonferroni correction for post-hoc analysis of the radiographic variables

Variables	CDI	BP	MIS
Native–pre-TO	***0.02***	***0.02***	***0.02***
Native–post-TO	0.59	0.59	***0.02***
Native–last	***0.001***	***0.001***	***< 0.001***
Pre-TO–post-TO	0.72	0.72	1.00
Pre-TO–last	1.00	1.00	0.06
Post-TO– last	***0.048***	***0.048***	***0.048***

Significant differences between two variables are displayed in **bold and italic**

*CDI* Caton-Deschamps index, *BP* Blackburne-Peel ratio, *MIS* modified Insall-Salvati index, *native* before total knee arthroplasty, *pre-TO* immediately before tuberositas osteotomy, *post-TO* immediately after tuberositas osteotomy, *last* final follow-up

### Correlations between clinical outcomes and radiographic variables

There was a moderate correlation between the last ROM and last BP (*r* = 0.35, *p* = 0.01) and a strong correlation between the pre-TO WOMAC and pre-TO MIS (r = 0.64, *p* = 0.04) (Table 5). There were no other correlations between clinical outcomes and radiographic variables.

**Table 5:** Correlations between clinical outcomes and radiographic variables

Variables	Pre-TO CDI	Last CDI	Pre-TO BP	Last BP	Pre-TO MIS	Last MIS
Pre-TO ROM	0.08 (0.54)	/	0.11 (0.43)	/	0.10 (0.50)	/
Last ROM	/	0.23 (0.08)	***/***	***0.35 (0.01)***	/	0.23 (0.07)
Pre-TO KSS Knee Score	0.10 (0.70)	/	0.23 (0.36)	/	0.21 (0.41)	/
Last KSS Knee Score	/	0.05 (0.79)	/	0.20 (0.25)	/	−0.05 (0.76)
Pre-TO KSS Function Score	−0.06 (0.83)		−0.05 (0.84)	/	−0.00 (1.00)	/
Last KSS Function Score	/	−0.03 (0.86)	/	0.12 (0.50)	/	−0.01 (0.97)
Pre-TO WOMAC	0.21 (0.54)	/	0.16 (0.63)	/	***0.64 (0.04)***	/
Last WOMAC	/	−0.21 (0.23)	/	−0.30 (0.09)	/	−0.06 (0.75)

Data are presented as Spearman correlation coefficient (*p* value)

Significant correlations are displayed in **bold and italic**

*CDI* Caton-Deschamps index, *BP* Blackburne-Peel ratio, *MIS* modified Insall-Salvati index, *ROM* range of motion, *KSS* Knee Society Score, *native* before total knee arthroplasty, *pre-TO* immediately before tuberositas osteotomy, *post-TO* immediately after tuberositas osteotomy, *last* final follow-up

### Interobserver reliability

The interobserver reliability was perfect, ranging from 0.86 (for the BP) to 0.93 (for the MIS).

## Discussion

Although some previous studies reported that proximal transfer of the tibial tubercle presents a satisfactory solution for *patella baja* after TKA [4, 5, 14, 15], these findings were not confirmed in our retrospective long-term study of 60 patients. We demonstrated with perfect interobserver reliability that proximalisation of the tibial tubercle did not lead to a significant improvement and even led to deterioration of the clinical scores (KSS and WOMAC) after a mean follow-up of almost five years. Despite the ROM improving significantly after the intervention, the mean postoperative total active ROM was 88°, which was far from satisfactory. Additionally, the radiographic variables (CDI, BP, and MIS) did not significantly improve after proximalisation of the tibial tubercle and even significantly decreased during long-term follow-up.

Whereas some studies have reported that proximalisation of the tibial tubercle achieved pain relief [8, 15] and functional improvement [8, 14, 15] in mid-term follow-up, our study did not confirm these findings, as both the KSS and WOMAC did not improve after proximal transfer of the tibial tubercle. A retrospective study found that 13 patients who underwent proximal tibial tubercle transfer for pseudo-*patella baja* after TKA had a significantly greater increase in the KSS at 2 years postoperatively than a control cohort that underwent revision surgery without proximalisation, especially for patients with proximalisation of more than 1 cm [14]. However, compared with our cohort, this previous study cohort was significantly younger (mean age 56 years vs. 61 years), was followed up for only two years after the intervention, and had a lower average preoperative BP (0.29 vs. 0.61). Furthermore, the patients in the previous study showed an improvement in the KSS Knee Score from < 30 to > 70 and an improvement in the KSS Function Score from 20 to > 50, whereas our patients already had higher scores prior to the proximalisation and achieved a higher mean KSS Function Score after the intervention. Therefore, our patients might have had limited potential for improvement compared with the other study cohort, which again is neither significant nor relevant in clinical practice. Another study investigated the functional improvement in 21 patients with *patella baja* after TKA and found a significant improvement in the mean KSS Knee Score and Function Score from preoperative values of 58 and 40, respectively, to postoperative values of 88 and 80, respectively [15]. While the mean age and BMI of this previous cohort were comparable to ours, the follow-up was less than three years, which was much shorter than our follow-up of five years in which further functional deterioration is possible. Moreover, the previous cohort had a very much lower average KSS Function Score prior to the intervention compared with our cohort (40 vs. 65); thus, this previous cohort might also have had greater potential to improve [15].

Our WOMAC results also contrast with the literature. Another study that investigated the efficacy of proximal tibial tubercle displacement in 15 patients with *patella baja* in the native joint reported significant improvements in the WOMAC and VAS pain score after a mean follow-up of approximately five years [8], whereas our patients showed a decrease in the WOMAC after the same follow-up duration. Again, the patients in this previous study were slightly younger and had a poorer BP prior to proximalisation compared with our cohort (0.4 vs. 0.61) and hence may have had greater potential for improvement. Furthermore, the aforementioned study included a small cohort with vast variations in underlying pathologies and causes of *patella baja*, without any patients with *patella baja* after TKA.

Our findings demonstrated a significant improvement in the ROM after proximal transfer of the tibial tubercle; however, we do not consider this relevant in clinical practice, as our patients did not even achieve an average post-intervention ROM of 110°, which is considered the minimum ROM required for activities in normal daily life [13]. The abovementioned study reported a smaller mean ROM in the 15 patients with *patella baja* in the native joint prior to proximalisation of the tibial tubercle (70° flexion range with 20° extension lag) and a larger postoperative flexion range (110° flexion range, with half of the patients still having an extension lag between 5° and 10°) compared with our cohort [8]. Similarly, the study by Vives-Barquiel also documented a mean preoperative ROM of 70° and significant improvement to an average of 100° after proximalisation of the tibial tubercle in 21 patients with* patella baja* after TKA [15]. As mentioned above, the cohorts in both previous studies were not comparable to our cohort due to the younger age, shorter follow-up, different underlying pathologies, and greater potential for improvement. An older study of 26 patients who underwent osteotomy of the tibial tubercle in conjunction with TKA reported a similar improvement in postoperative ROM to our study; after a mean follow-up of three years, the mean ROM was 77° [17]. However, in this previous study, proximal transfer of the tibial tubercle was performed to gain surgical exposure during joint replacement, not due to underlying *patella baja* [17].

The radiographic findings of the course of the CDI, BP, and MIS confirmed our hypothesis that proximalisation of the tibial tubercle initially leads to a non-significant restoration of patellar height, which significantly decreases again over time to return to or even become lower than the preoperative level. Both abovementioned studies documented a radiographic normalisation of the patellar height at three and five years after the intervention [8, 15]. However, one study investigated the situation at only three years postoperatively and the other did not include patients with joint replacement. To the best of our knowledge, the present study was the first to examine the change in patellar height during long-term follow-up after proximal transfer of the tibial tubercle, and confirmed the suspected de novo decline in patellar height after an initial non-significant improvement. Consequentially, our findings raise awareness that there is no need to perform proximalisation of the tibial tubercle to restore patellar height in patients with *patella baja* after TKA, as this intervention does not seem to be effective in the long-term.

Finally, we observed few correlations between clinical outcomes and radiographic variables prior to proximal transfer of the tibial tubercle, except for a moderate correlation between the last ROM and BP and a strong correlation between the pre-TO WOMAC and MIS. Vives-Barquiel et al. also found a positive correlation between ROM and BP [15]; however, the strength of the correlation was not specified. No other study has reported a strong correlation between the preoperative WOMAC and MIS.

The strength of our study is that it presents the first long-term follow-up study of clinical function and various radiographic variables in a cohort of 60 patients with *patella baja* after TKA that underwent proximal transfer of the tibial tubercle. However, in addition to its retrospective design, our study has several limitations. First, although we were able to measure radiographic variables at various timepoints, the retrospective design meant that post-TO clinical scores were not available. Hence, we cannot assess the short-term clinical outcomes of proximal transfer of the tibial tubercle to evaluate whether patients had better knee function immediately after the intervention (when the radiographic variables were improved). Furthermore, our investigation constitutes a non-comparative retrospective analysis without a control group. As patients with *patella baja* after TKA who are scheduled for revision surgery at our institution generally undergo proximalisation of the tibial tubercle, an appropriate control group would include patients who received conservative treatment of *patella baja*. Additionally, this retrospective analysis cannot define the reasons for the deterioration of radiographic patellar height and unsatisfactory clinical outcomes; these reasons may include biomechanical factors such as the height of the joint line, the tibial slope, or under- and overstuffing of prosthetic components; further studies are needed to investigate the reasons for the unfavourable outcomes. Finally, there may be bias due to the underlying heterogeneity of the subjects with a relevant percentage undergoing revision surgery due to infection, as septic knee revisions result in worse function compared to revisions for non-infectious reasons. Additionally, in some cases of *patella baja*, tibial tubercle transfer was not the first treatment attempt and some patients underwent other salvage procedures as closed manipulation under anaesthesia before proximalisation of tibial tubercle. Hence, concomitant procedures might bias the outcome after tibial tubercle transfer in those patients.

## Conclusions

Proximalisation of the tibial tubercle in patients with *patella baja* does neither lead to a significant improvement in the clinical outcomes (KSS and WOMAC) nor in the radiographic patellar height during long-term follow-up, as the CDI, BP, and MIS significantly decreased during 5 years of follow-up after the intervention. Proximal tibial tubercle transfer only significantly improves the knee ROM. However, the postoperative ROM does not reach the minimum ROM required for activities in normal daily life. Therefore, the indication for proximal tibial tubercle transfer for* patella baja* should include a thorough analysis of all reasons for this clinical and radiographic pathology.

## Data Availability

The analysed dataset of this investigation is available from the corresponding author on reasonable request.
